# Intraparenchymal papillary meningioma of brainstem: case report and literature review

**DOI:** 10.1186/1477-7819-10-10

**Published:** 2012-01-12

**Authors:** Xiao-Bing Jiang, Chao Ke, Zhi-An Han, Shao-Hua Lin, Yong-Gao Mou, Rong-Zhen Luo, Shao-Xiong Wu, Zhong-Ping Chen

**Affiliations:** 1 Department of Neurosurgery, Sun Yat-sen University Cancer Center, No. 651, Dong Feng Road E., Guang-zhou 510060, P. R. China; 2 Department of Neurosurgery, Zhongshan People's Hospital, No.2, Sun Wen Road E., Zhong-shan 528403, P. R. China; 3 Department of Pathology, Sun Yat-sen University Cancer Center, No. 651, Dong Feng Road E., Guang-zhou 510060, P. R. China; 4 Department of Radiation Oncology, Sun Yat-sen University Cancer Center, No. 651, Dong Feng Road E., Guang-zhou 510060, P. R. China

**Keywords:** Meningioma, Papillary, Brain stem, Intraparenchymal, Cyst

## Abstract

Both intraparenchymal papillary meningioma and papillary meningioma with cyst formation of brainstem have never been reported. The authors present an extremely rare case of patient with intraparenchymal papillary meningioma of brainstem. A 23-year-old Chinese male presented with a 4-month history of progressive left upper limb and facial nerve palsy. Magnetic resonance imaging revealed a cystic-solid, heterogeneously enhancing mass in pons and right cerebral peduncle with no dural attachment. The tumor was totally removed via subtemporal approach. During surgery, the lesion was found to be completely intraparenchymal. Histological and immunohistochemical examinations were compatible with the diagnosis of papillary meningioma. The lesion recurred nine months after primary surgery, a second surgery followed by radiotherapy was performed. Till to now (nearly 2 years after the treatment), the patient is tumor free survival. Intraparenchymal meningioma of brainstem with cystic formation is very rare, however, it should be considered as a differential diagnosis of a brainstem neoplasm. The present case strongly recommended that postoperative radiotherapy was essential for the patients with papillary meningiomas.

## Background

Meningiomas account for nearly 35% of all primary intracranial neoplasms, deriving from the arachnoid cap or meningothelial cells [1]. Therefore, they usually display a dural attachment [2]. Unusually, they could present with no dural attachment [3-6]. The majority of the meningiomas without dural attachment are supratentorial, whereas the infratentorial ones are relatively less frequent. In reviewing the literatures, only thirteen infratentorial meningiomas without dural attachment excluding the one in the fourth ventricle have been reported, and only one case was in the brainstem [7]. Intraparenchymal papillary meningiomas of brainstem, to our knowledge, have never been reported before.

Cyst formation is uncommon in the meningioma, which most commonly develops in fibroblastic and meningothelial meningiomas. Papillary meningiomas demonstrating cystic changes were reported only in five cases in previous literatures [8]. Here, we report a case of intraparenchymal meningioma of brainstem. This should be the first case of intraparenchymal papillary meningioma of brainstem, as well as the first papillary meningioma with cyst formation located in brainstem.

## Case presentation

**Figure 1:**
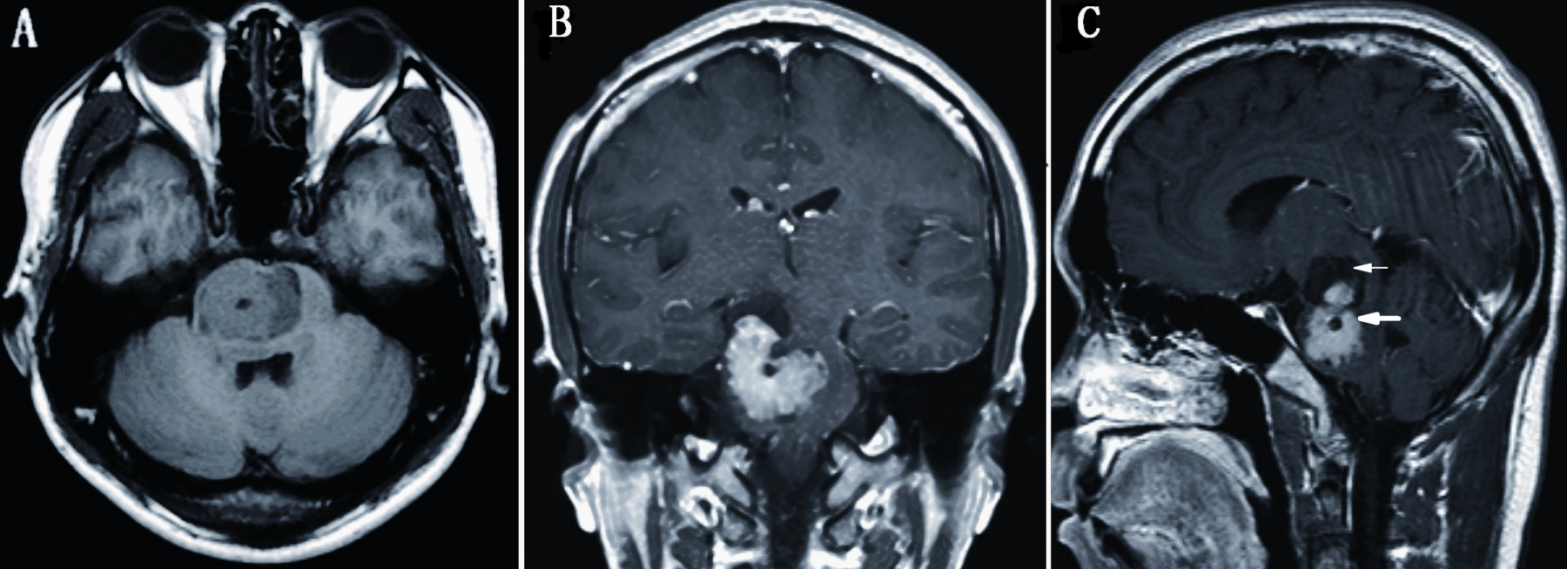
**Preoperative magnetic resonance imaging demonstrating a cystic-solid mass of brainstem with mild edema**. The solid part manifested homogeneous enhancement on T1-weighted gadolinium enhancement (thick arrow). The well-defined mass was mainly located in pons with the cystic part (thin arrow) extending to the right cerebral peduncle, but there was no evidence of attachment to dura mater. Axial T1-weighted imaging (A), coronal (B) and sagittal (C) post-contrast T1-weighted imaging.

A 23-year-old Chinese male presented with a 4-month history of progressive left upper limb and facial nerve palsy. In the month prior to admission, he was suffering from impairment of the left upper limb movement, as well as mild dysphasia. Magnetic resonance imaging (MRI) revealed a cystic-solid mass, sized 35*25*20 mm, located in the brainstem. The solid part of the tumor mainly located in the pons, while the cystic part extended to the right cerebral peduncle. The solid part manifested iso-intense on both T1- and T2- weighted imaging with homogeneous enhancement on T1-weighted gadolinium enhancement, but no dural tail sign was noted. The cystic part was shown of hypo-intense on T1-weighted imaging and hyper-intense on T2-weighted imaging (Figure 1).

The patient underwent a total surgical resection of the tumor mass via a right subtemporal approach, under general anesthesia. The cystic-like part of the mass was full of xanthochromic fluid, and the solid part was found to be fragile and hemorrhagic, with defined margin from the adjacent tissue. The lesion was purely intraparenchymal, and no dural or leptmeninges attachment was observed during the operation. The tumor was removed totally, the postoperative course was uneventful. His left side facial palsy was completely relieved and his left upper limb returned to be normal after operation.

**Figure 2:**
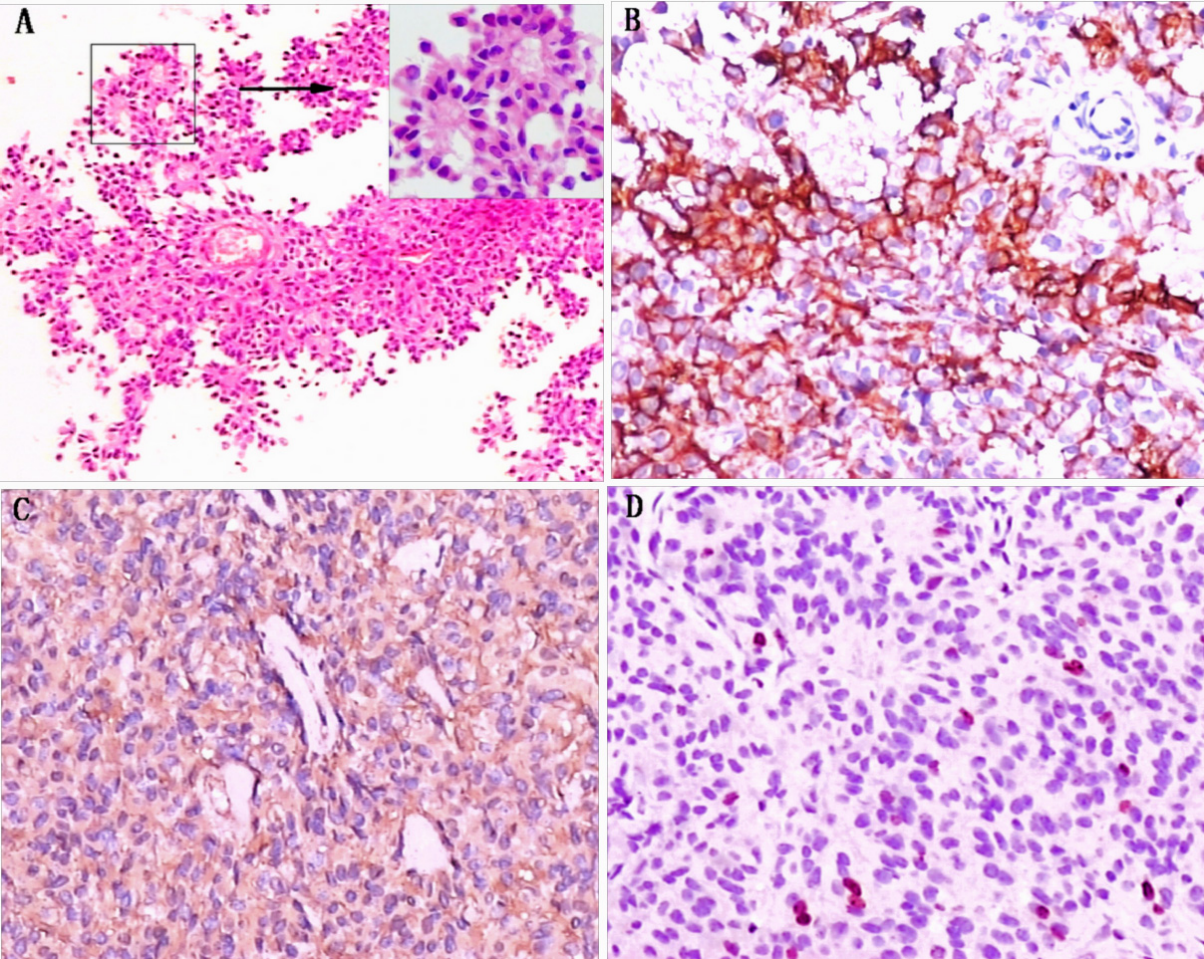
**Photomicrographs showing the histological and immunohistochemical features of the lesion**. The tumor tissue was composed of sheets of cells arranged in a predominantly papillary pattern with perivascular pseudo-rosettes. Tumor cells in the papillary area displayed abundant eosinophilic cytoplasm, vesicular nuclei (A) (original magnification ×100). The immunohistochemistry profile showed positivity for EMA (B), vimentin (C) and MIB-1 (D) (original magnification ×200). The insert in A represents a higher magnification of the outlined area (original magnification ×400).

The histological examination revealed a tumor tissue composed of sheets of cells arranged in a predominantly papillary pattern with perivascular pseudo-rosettes. Tumor cells in the papillary area displayed abundant eosinophilic cytoplasm, vesicular nuclei but few mitoses. The mean of MIB-1 labeling index was about 3%. The immunohistochemistry profile showed positivity for epithelial membrane antigen (EMA) and vimentin, but negativity for glial fibrillary acidic protein (GFAP), cytokeratin (CK), and S-100 protein (Figure 2). These findings were consistent with papillary meningioma (World Health Organization grade III, WHO III). The patient was recommended for radiotherapy, but he refused to further treatment.

**Figure 3:**
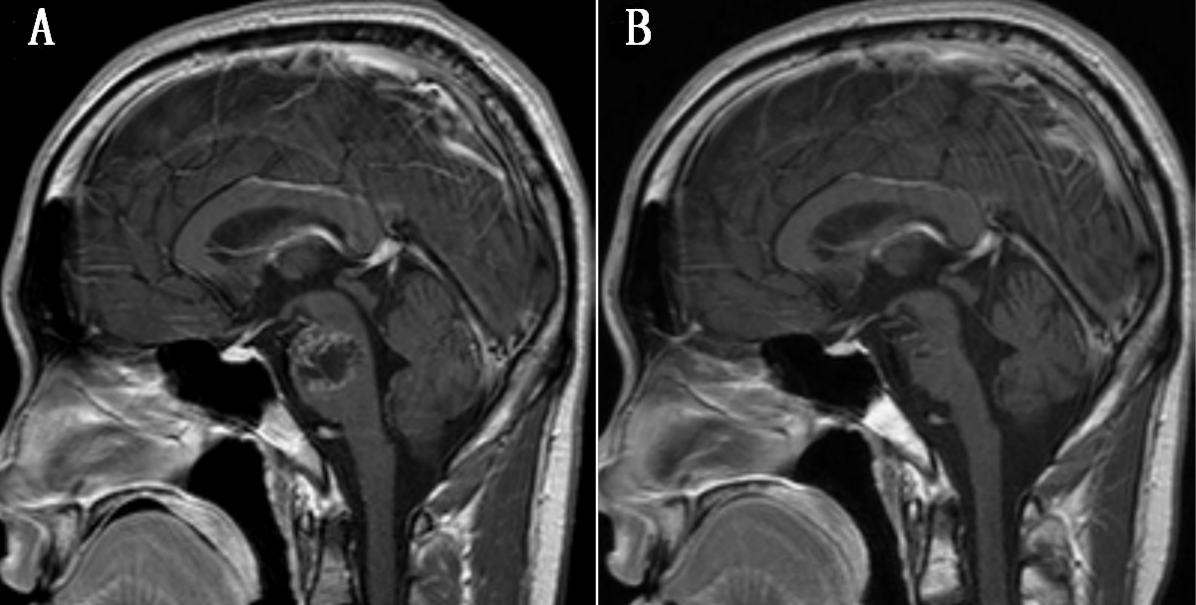
**Nine-month after the first operation, magnetic resonance imaging revealed a local recurrence of primary tumor, which showed heterogeneous enhancement on post contrast imaging (A)**. Two-year after secondary surgery plus radiotherapy. T1-weighted enhanced magnetic resonance imaging shows no evidence of disease residue or recurrence (B).

Nine months later, he was re-admitted with facial palsy and left upper limb weakness again, and brain MRI showed local tumor recurrence (Figure 3A). He was re-operated on through the previous approach, with a total tumor resection. Histological analysis confirmed the diagnosis of papillary meningioma again. One month later, he received local external radiotherapy. During the more than 2 years follow-up, brain MRI revealed no residual or recurred tumor (Figure 3B). But his left upper limb weakness remained grade IV despite rehabilitation therapy.

## Discussion

Infratentorial meningiomas without dura attachment are uncommon. Reviewing the literatures [9-12], only forty-four cases have been reported including thirty-one intraventricular meningiomas. In previous literatures [6,13,14], infratentorial meningiomas without dural attachment were classified into 5 types: meningioma arising from the choroid of the fourth ventricle and lying wholly within it, meningioma arising from the inferior tela and lying partially in the fourth ventricle and partially in the cerebellar hemisphere, meningioma lying in the cisterna magna, meningioma arising from the choroid plexus and lying in the lateral cerebellomedullary cistern, and intraparenchymal meningioma. However, Zhang et al. [6] suggested that meningiomas in the ventricular system should be distinguished from the other types of no dural attachment meningiomas, because they were quite different from each other. Intraparenchymal meningioma is considered as a same type of subcortical meningioma, which locates in brain parenchyma without dural attachment, although they can reach the surface of the brain [6,15]. Based on the imaging and intraoperative findings, the present case should be classified as an intraparenchymal meningioma. To the best of our knowledge, there was only one case of intraparenchymal brainstem meningioma reported [7], who was a 22-month old child with a clear cell meningioma located in the upper medulla and pons. Similarly, the lesion in present case mainly located in pons and extended to cerebral peduncle. Nevertheless, intraparenchymal papillary meningioma of brainstem has never been described before. Herein, including present case, only fourteen cases of infratentorial meningiomas without dural attachment have been reported in the literatures, except for the one in the fourth ventricle. There were four cases deriving from the inferior tela choroidea [16], four cases lying in the cisterna magna [16,17], four cases in the cerebellomedullary cistern [13,18-20], and two cases were intraparenchymal [7].

Clinical characteristics of eight infratentorial meningiomas without dural attachment were summarized in table 1. Four cases from the inferior tela choroidea and two cases lying in the cisterna magna were excluded from the clinical data, as we couldn't found integrated data in the literatures. The average age of the 8 patients at surgery was 42.5 years (range, 1.8-72 years), which was similar to the one in the fourth ventricle (45 years) [9], but much older than that of deep sylvian meningiomas (25.8 years), pineal region meningiomas (33.1 years) without dural attachment and supratentorial intraparenchymal meningiomas (15.4 years) [4,6]. It might remind us that the patients with infratentorial meningiomas without dual attachment are prone to be older adults, comparing to those with supratentorial ones. However, they all presented similar male to female ratio, which was a sharp contrast to the female predilection of general meningiomas and meningiomas in the fourth ventricle [4,6,9]. In consistent with the other types of meningiomas, fibroblastic and meningothelial meningiomas were the most common types, but the ratio of WHO grade II and III (25%) seemed to be higher. While, only 8 cases limited our ability to reach a firm conclusion.

**Table 1:** Summary of published infratentorial meningiomas without dural attachment excluding the one in the fourth ventricle (n = 8)

Author	Age (ys.)	Sex	Symptoms	Location	Computer tomography	Magnetic resonance	Size(mm)	Surgical removal	Complications	Histology
Kim et al.	59	F	Headache, dizziness	Left cerebellum-edullary cistern	Not reported	T1., isointense T2., heterogeneous C.e.,homogenous enhancement	52 × 28	Subtotal*	Mild and transient dysphasia	Mixed pattern of meningothelial and fibroblastic
Shibuya et al.	61	F	Unsteady gait	Left cerebellom-edullary cistern	Round enhancing mass with multiple foci of calcification	T1., isointense T2., high intense C.e., heterogeneous enhancement	40	Subtotal	Transient dysphasia	Meningothelial
Ishigaki et al.	14	M	Dizziness	From right CPA to the foramen of Luschka	High density without calcification	T1., isointense T2., high intense C.e., heterogeneous enhancement	30	Total	Husky voice	Meningothelial
Nakahara et al.	56	M	None	Lateral site of right cerebellar cortex	Small round non-enhancing lesion	T1., hypointense T2., not reported C.e., partial enhancement	Not reported	Total	None	Fibroblastic
Teo et al.	1.8	F	right hemipares-is, facial nerve palsy and dysphagia	Upper medulla and pons	Not reported	T1., not reported T2., not reported C.e., multilobulated mass	40 × 40 × 38	Partial	Persistent right hemiparesis with right facial nerve palsy	Clear cell
Nicoletti et al.	53	M	Nausea and vomiting	Cisterna magna	Not reported	T1., isointense T2., not reported C.e., homogeneous enhancement	About 35 × 27	Total	None	Meningothelial
Jung et al.	72	F	Neck pain and a tingling sensation of left arm	Cisterna magna	Not reported	T1., isointense T2., hyperintense C.e., homogeneous enhancement	33 × 20 × 20	Total	None	Syncytial and fibroblastic
Present case	23	M	Left upper limb nerve palsy and impairment of fine movement	Pons and right cerebral peduncle	Not performed	T1., heterogeneous T2., heterogeneous C.e., cystic-solid mass with heterogen- eous enhancement	35 × 25 × 20	Total	Mild left upper hemiparesis	Papillary

Ys., years. M, male, F, female. CPA, cerebellopontine angle. T1., T1-weighted imaging, T2., T2-weighted imaging, C.e., contrast enhancement. * With postoperative radiotherapy.

Infratentorial meningiomas without dural attachment represent surgical challenge, requiring special considerations, because of the vicinity of the brainstem, cranial nerves, and veterbrobasilar arteries. Despite recent improvements in surgical techniques, surgical treatment of meningiomas in this area still carries a high risk of morbidity and mortality. In some cases, the lesion might be strongly adherent to these structures, leading to a partial or subtotal resection. Nevertheless, in more than half of the reports (5/8), a total resection was reached with mild complications. This might be largely ascribed to the distinct relationship between lesion and surrounding tissues.

Papillary meningioma is an aggressive variant of meningioma, which accounts for 1-2.5% of all meningiomas [21]. In comparing with benign meningiomas, they usually display aggressive clinical behavior marked by a high rate of brain parenchymal invasion, local recurrence, and extracranial metastases. It is pathologically defined by the presence of perivascular pseudopapillary pattern either entirely or more commonly in combination with other common histological componets of meningiomas. It has been graded as a WHO grade III tumor [22,23]. Cyst formation is uncommon in meningiomas, which most commonly develops in fibroblastic and meningothelial type meningiomas. The mechanisms leading to cyst formation are still unclear. Confluence of micro-cysts and intratumoral hemorrhages may be possibilities regarding the formation of intratumoral cysts [24]. Only five cases of papillary meningioma with cyst formation were reported in the literatures [8]. The present case should be the first description of intraparenchymal papillary meningioma with cyst formation arising in brainstem. The presence of cyst formation and heterogeneous enhancement made it mimic other neoplasms, such as gliomas, hemangioblastomas, and metastatic tumors. Nevertheless, a remarkable papillary pattern of tumor tissue, as well as immunohistochemistry findings, strongly support the fact that it is a papillary meningioma. The lesion recurred in only nine months despite total resection of primary tumor, which was also in accordance with the aggressive clinical behavior of papillary meningioma.

Due to the rarity of papillary meningiomas, no clear consensus has achieved regarding the proper management of papillary meningiomas. However, aggressive surgical resection plus postoperative radiotherapy has come to represent the standard care [20]. In current case, both of the primary and recurrent lesions were totally removed with mild complications. If the patient underwent postoperative radiotherapy, recurrent of primary tumor might not occur in such short period. Fortunately, the patient remains free from tumor more than 2 years after repeated resection plus radiotherapy.

## Conclusions

The authors described an extremely unusual case with intraparenchymal papillary meningioma of brainstem with cyst formation. Although this is very infrequent, meningiomas should be included in the differential diagnosis of an intraparenchymal brainstem tumor. Papillary meningioma is a distinct variant of meningioma with highly aggressive behavior. The experience of the present case strongly indicates that postoperative radiotherapy is essential for the patients with papillary meningiomas.

### Consent

Written informed consent was obtained from the patient for publication of this Case report and any accompanying images. A copy of the written consent is available for review by the Editor-in-Chief of this journal.

## Competing interests

The authors declare that they have no competing interests.

## Authors' contributions

XJ and CK participated in the study design. All authors participated in the treatment applied and data collection. XJ and ZC participated in literature reviewing and manuscript writing. XJ, ZC and CK participated in editing and proof reading. All authors read and approved the final manuscript.
